# The circadian clock in the choroid plexus drives rhythms in multiple cellular processes under the control of the suprachiasmatic nucleus

**DOI:** 10.1186/s12987-024-00547-3

**Published:** 2024-05-27

**Authors:** Martin Sládek, Pavel Houdek, Jihwan Myung, Kateryna Semenovykh, Tereza Dočkal, Alena Sumová

**Affiliations:** 1 Laboratory of Biological Rhythms, Institute of Physiology of the Czech Academy of Sciences, Videnska 1083, Prague 4, 14200 Czech Republic; 2 Graduate Institute of Mind, Brain and Consciousness (GIMBC), Taipei Medical University, Taipei, Taiwan; 3 Brain and Consciousness Research Centre (BCRC), TMU-Shuang Ho Hospital, New Taipei City, Taiwan

**Keywords:** Choroid plexus, Circadian clock, Circadian transcriptome, Mouse, Suprachiasmatic nuclei, *mPer2*^*Luc*^ mouse, Glucocorticoid

## Abstract

Choroid plexus (ChP), the brain structure primarily responsible for cerebrospinal fluid production, contains a robust circadian clock, whose role remains to be elucidated. The aim of our study was to [1] identify rhythmically controlled cellular processes in the mouse ChP and [2] assess the role and nature of signals derived from the master clock in the suprachiasmatic nuclei (SCN) that control ChP rhythms. To accomplish this goal, we used various mouse models (WT, *mPer2*^*Luc*^, ChP-specific *Bmal1* knockout) and combined multiple experimental approaches, including surgical lesion of the SCN (SCNx), time-resolved transcriptomics, and single cell luminescence microscopy. In ChP of control (Ctrl) mice collected every 4 h over 2 circadian cycles in darkness, we found that the ChP clock regulates many processes, including the cerebrospinal fluid circadian secretome, precisely times endoplasmic reticulum stress response, and controls genes involved in neurodegenerative diseases (Alzheimer’s disease, Huntington’s disease, and frontotemporal dementia). In ChP of SCNx mice, the rhythmicity detected in vivo and ex vivo was severely dampened to a comparable extent as in mice with ChP-specific *Bmal1* knockout, and the dampened cellular rhythms were restored by daily injections of dexamethasone in mice. Our data demonstrate that the ChP clock controls tissue-specific gene expression and is strongly dependent on the presence of a functional connection with the SCN. The results may contribute to the search for a novel link between ChP clock disruption and impaired brain health.

**Supplementary Information:**

The online version contains supplementary material available at 10.1186/s12987-024-00547-3.

## Introduction

Choroid plexus (ChP) is a non-neural structure in the brain ventricles [1–3] that has recently attracted attention because of its multiple roles in brain homeostasis. Apart from its well-recognized functions in the production of most cerebrospinal fluid (CSF) [4, 5] and the formation of the blood-CSF barrier [6], it has other functions. These include the production of bioactive molecules, participation in metabolite clearance, formation of a toxin barrier, and critical roles in brain metabolism, neurosignaling, neuroinflammatory processes, and neuroprotection (reviewed in [7]). Malfunction of these processes is believed to be associated with poor brain health and the development of neurodegenerative disorders [8, 9].

Brain functions continuously respond to changes in both the external and internal environments, which occur in a daily cycle. As an adaptation, various cerebral processes are controlled rhythmically in synchrony with the day-night cycle by endogenous clocks [10]. These clocks generate rhythmic signals with approximately 24-hour (circadian) period via a mechanism based on a cellular transcriptional and translational feedback loop (TTFL, reviewed in [11]). The clock mechanism results in the rhythmic expression of families of clock genes (such as *Per1-3, Cry1-2, Nr1d1-2, Rora-c, Bmal1, Clock* and *Npas2*) [12]. Protein products of these genes serve as transcriptional activators or repressors that govern the expression of a wide range of downstream clock-controlled genes. These genes, in turn, fulfill pivotal roles in tissue-specific rhythmic processes [13].

Although the clock mechanism operates endogenously in almost every cell in the body, there is only one structure in mammals that houses the “central” clock. This clock is uniquely able to respond directly to information from the retina about changes in the external light/dark cycle, which reflects the time of day. It is located in the suprachiasmatic nuclei of the hypothalamus (SCN, reviewed in [14, 15]) and sends rhythmic output signals to clocks in other parts of the brain and the rest of the body to maintain their appropriate phases [16]. Moreover, signals from the SCN can drive rhythmicity in regions with weak coupling among oscillatory cells typically represented by most areas in the brain [17].

ChP is a rare example of an extra-SCN brain region with a robust circadian clock [18]. Still, its involvement in regulating ChP physiological functions remains a matter of speculation (reviewed in [19]). The clock maintains its rhythmicity when ChP is explanted in an in vitro culture, i.e., conditions lacking any signal from the SCN [18, 20, 21], demonstrating its self-sustainability under artificial conditions. Nevertheless, the clock is highly sensitive to external SCN-controlled signals such as glucocorticoids; dexamethasone affected ChP clock in vivo, in rats with adrenalectomy, and in organotypic mouse explants cultured in vitro, where the effect was blocked by the specific antagonist mifepristone [21]. Dexamethasone increased the amplitude and reset ChP clock via intracellular pathways that depend on functional PKA-ERK1/2 signaling [21]. Moreover, the effects of pharmacological manipulations of GSK3 activity and lithium-responsive signaling pathways suggested another potential mechanism providing input for entrainment of ChP clock [22]. These results provide evidence that ChP can sense and respond to external signals that modulate canonical intracellular signaling pathways, which converge to the circadian clock.

Consistent with other processes in the brain, CSF production in humans is subject to daily fluctuations with a peak in production at night, i.e., during sleep [23, 24], although the underlying mechanism remains to be elucidated [25]. A ChP-specific transcriptome and proteome have been described [26–31]. Moreover, a recent study demonstrated differences in ChP proteome by comparing samples collected at two time points, 7 am and 7 pm [32]. However, the circadian (i.e., endogenously driven) changes in ChP transcriptome and their dependence on the SCN clock have not been studied. Furthermore, the role and nature of SCN signaling regulating the circadian clock in the ChP are unknown. To address this gap, we performed a time-resolved transcriptome analysis of ChP samples collected over two days in constant darkness from mice with intact or surgically removed SCN, complemented by real-time recordings of explanted ChP at single-cell resolution. Analyses of the data revealed extensive rhythmicity in ChP of control mice, thus providing the first evidence for circadian control of multiple ChP functions. Additionally, our results showed a high dependence of the rhythms on the central clock in the SCN; an SCN lesion resulted in an almost complete loss of ChP rhythms detectable in vivo and ex vivo. This effect was comparable to that of genetic knockout specifically of the ChP clock. We identified the glucocorticoid signaling as one of the possible pathways used by the SCN to entrain the ChP clock because repeated daily injections of dexamethasone to SCN-lesioned mice restored ChP clock rhythmicity.

## Materials and methods

### Animals

Animal experimentation was approved by the Animal Care and Use Committee of the Institute of Physiology (33/2019) and by Taipei Medical University (LAC-2020-0576). It was performed in accordance with the Animal Protection Law of the Czech Republic as well as the European Community Council directives 2010/63/EU, and the Animal Protection Act of Taiwan. All efforts were made to reduce the suffering of the animals. Adult male C57BL/6J mice (Charles River, Germany) (4- to 6-month-old) and knock-in *mPer2*^*Luc*^ male mice [33] on C57BL/6J background (strain B6.129S6-*Per2*^*tm1Jt*^*/J, JAX, USA;* a colony maintained at the Institute of Physiology, the Czech Academy of Sciences) (5- to 8-month-old) were housed individually in a temperature-controlled facility at 23±2 °C with free access to food and water. The light/dark cycle was maintained with 12 h of light and 12 h of darkness (LD12:12); lights were turned on and off at 06:00 and 18:00, respectively. Prior to sampling, C57BL/6J and *mPer2*^*Luc*^ mice were kept in constant darkness. Tissue-specific knockout FoxJ1-ERT2-Cre^+/−^ (JAX stock #027012) [34] and *Bmal1*^fl/fl^ mice (JAX stock #007668) [35], both on C57BL/6J background, were crossbred at the Taipei Medical University Laboratory Animal Center to produce FoxJ1-ERT2-Cre^+/−^; *Bmal1*^fl/fl^ mice. Tamoxifen was provided ad libitum through the diet (tamoxifen citrate 0.4 g/kg; Envigo TD.130,860) to 8-month-old male FoxJ1-ERT2-Cre^+/−^; *Bmal1*^fl/fl^ mice maintained under 12:12 LD cycle (07:00 light-on and 19:00 light-off) and controlled temperature (21.4 ± 0.2ºC) and humidity (59 ± 5%) conditions. The tamoxifen-induced flox recombination was tested in liver samples through qPCR (data not shown).

### Surgery

**Fig. 1:**
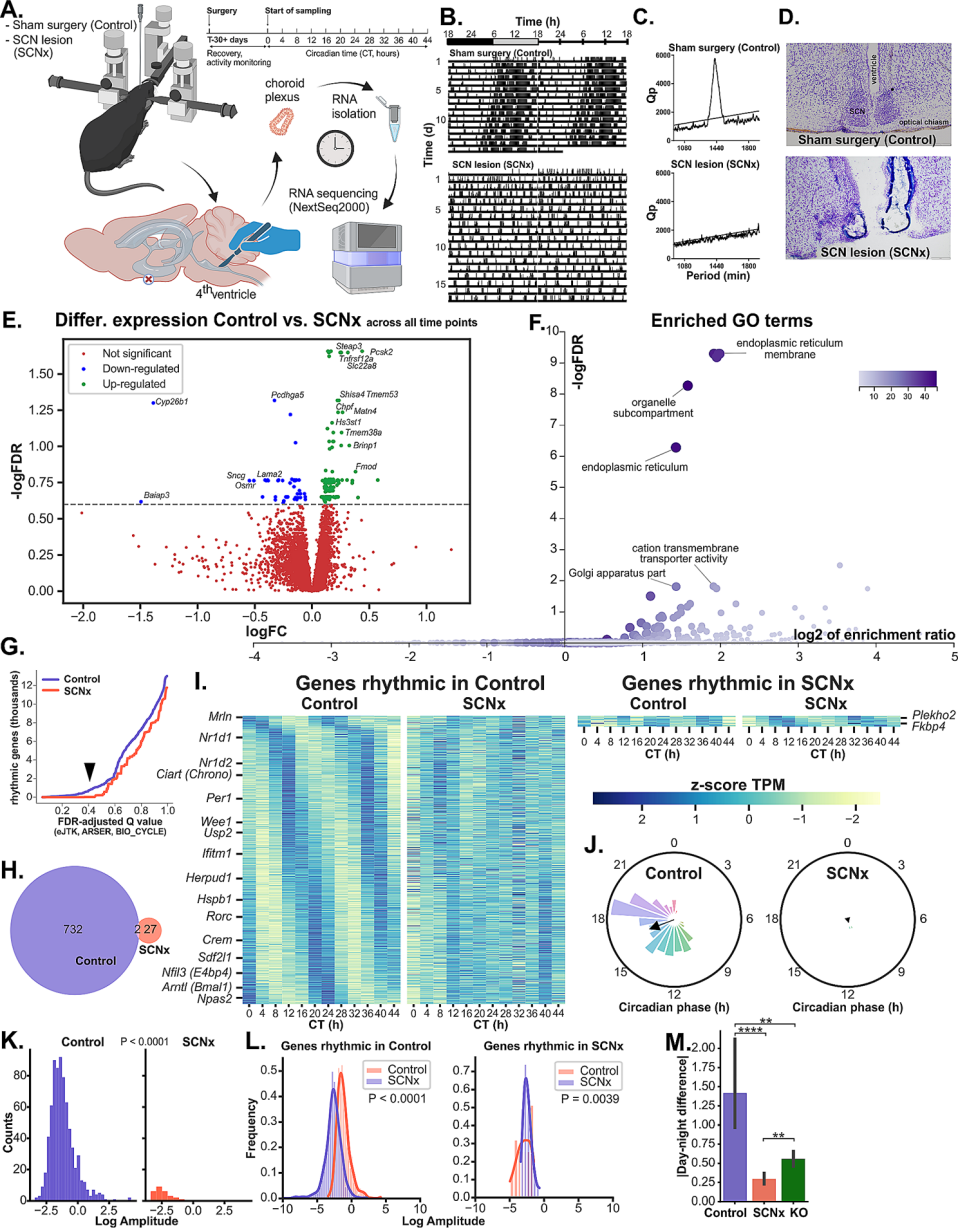
Suprachiasmatic nucleus lesion (SCNx) disrupts circadian transcriptome in choroid plexus (ChP) of the 4th ventricle, comparably to genetic disruption of local ChP clock. **(A)** Cartoon depicting the workflow; created with BioRender.com. **(B)** Representative double-plotted actogram of a mouse with sham surgery (controls) and SCN lesion (SCNx) recorded in constant darkness (DD); black and grey boxes indicate the time of lights off and on during the previous light-dark regime, respectively. **(C)** Corresponding Chi-square periodograms of the activity rhythms shown in Fig. 1B, Qp – Chi-square statistics. **(D)** Histology showing the SCN of a representative sham-operated mouse and of the SCNx mouse from Fig. 1B. **(E)** Volcano plot of genes identified as differentially expressed (DEGs) by pairwise DESeq2 analysis between Control and SCNx samples pooled across all time points using the dataset of genes with an average TPM ≥ 1 (referred to as filtered background). Selected genes are annotated; 174 up- (green) and downregulated (blue) genes submitted to further analysis are highlighted. **(F)** DEGs with Benjamini-Hochberg false discovery rate adjusted P value (FDR) < 0.25 were submitted to over-representation analysis (ORA) against filtered background. Volcano plot shows the most enriched Gene Ontology (GO) terms (annotated non-redundant terms). **(G)** The rhythmicity threshold (Q < 0.4) was chosen after plotting the number of cycling genes in Control (blue) and SCNx (red) against FDR Q value of three independent detection methods (eJTK, BIO_CYCLE, ARSER). **(H)** Venn diagram of genes identified as rhythmic in Control (red) and SCNx (green). **(I)** Heatmap of genes identified as significantly rhythmic in Control (left) or SCNx (right) samples, normalized and sorted by phase. Representative genes of distinct phase clusters are annotated. **(J)** Polar histogram of genes identified as rhythmic in Control (left) and SCNx (right) with calculated Rayleigh vector showing the mean phase. **K.** Histogram of log-transformed amplitudes of all genes identified as rhythmic in Control (left) and SCNx (right), Mann-Whitney test. **L.** Histograms of log-transformed amplitudes of all genes identified as rhythmic in Control and the same genes in SCNx (left), or vice versa (right), compared by Wilcoxon signed-rank test. **M.** Absolute values of normalized day-night differences of only the most cycling transcripts (Q < 0.1, *n* = 38) between the three groups (1 sample / time point). Dunn’s test, mean ± 95% CI (confidential interval), ** *P* < 0.01, **** *P* < 0.0001

C57BL/6J (for RNAseq) and *mPer2*^*Luc*^ (for circadian bioluminescence microscopy) mice were divided into two groups: the control group (Ctrl) was subjected to sham operation, and the other group was subjected to bilateral electrolytic SCN lesions (SCNx, Fig. 1A). The surgical ablation of the SCN was performed as previously described [36]. The mice were anesthetized with isoflurane inhalation (Isoflurin, Vetpharma Animal Health, Spain) and mounted in a stereotactic instrument (David Kopf Instruments, USA). The eyes were treated with the ointment Ophthalmo-Septonex (Zentiva, Czechia). The electrode tips (0.2 mm diameter) were placed bilaterally into the SCN through a drilled hole in the skull (coordinates: 0.1 mm rostral to bregma; ± 0.35 mm to midline; 5.55 mm below brain surface, and 0.1 mm caudal to bregma; ± 0.35 mm to midline; 5.65 mm below brain surface), and 0.6 mA current pulse was delivered for 6 s (53,500 Lesion Making Device, Ugo Basile, Italy). The parameters were determined empirically to achieve complete SCN lesion and avoid damage of surrounding tissue. Mice recovered in their home cages and received analgesic (NUROFEN, UK) immediately after the surgery (applied orally) and then in drinking water (2.5 ml per 250 ml) during the next day. The success of the SCN lesions (SCNx) was carefully checked (1) behaviorally by monitoring the locomotor activity in constant darkness for at least one week (Fig. 1B) and through periodogram analysis (Fig. 1C and Fig. S1), and (2) histologically postmortem for the absence of the SCN throughout the rostrocaudal brain sections of the anterior hypothalamus (Fig. 1D). The mice with detectable rhythm in locomotor activity in constant darkness and/or those in which histological examination of the hypothalamic sections revealed the presence of the SCN or its remaining parts were excluded from the study. The Ctrl group underwent sham surgery using the same procedure, including anesthesia and insertion of electrodes, but without the application of electric current. None of the sham-operated mice exhibited a disruption in circadian locomotor rhythmicity under constant darkness.

### Locomotor activity measurement

The locomotor activity of mice was monitored throughout the experiment individually in cages equipped with infrared movement detectors positioned centrally above the top of each cage, and the activity was detected using a circadian activity monitoring system (Dr. H.M. Cooper, INSERM, France) as required. The activity was recorded every minute, and double-plotted actograms and Chi-square periodograms [37] were generated to visualize the data with Chi-square statistics set at *P* < 0.0001; significant periodogram peaks within the circadian range of SCNx mice were below 10% of an average Ctrl peak (Fig. S1). The parameters of circadian rhythmicity were analyzed using the ClockLab toolbox (Actimetrics, USA) and Fiji ImageJ plugin ActogramJ [38].

### Collection of ChP samples for RNA isolation

After exposure to constant darkness to verify absence of locomotor activity rhythms as a marker of SCN lesion success, the C57BL/6J mice of Ctrl and SCNx groups (*n* = 12 per group) were returned to the original LD12:12 regime for one week to synchronize with the LD cycle. Subsequently, the lights were not switched on in the morning (assigned as circadian time, CT0), and mice remained in constant darkness for the following two days during which they were sacrificed every 4 h (from CT0 to CT44, 12 time points). ChP from the 4th ventricle was removed by pincers and immediately immersed into RNAlater. FoxJ1-ERT2-Cre^+/-^; *Bmal1*^fl/fl^ (KO) mice maintained in LD12:12 were sacrificed at two time points; 7 h after the lights on for the day time point, i.e., Zeitgeber time (ZT)7 and 6 h after the lights off for the night time point, i.e., ZT18, at Taipei Medical University, Taipei, Taiwan. Whole brains were removed, frozen on dry ice, embedded in OCT compound (Sakura Finetek), and then cold-shipped to Prague. Upon arrival, they were sectioned on cryocut and ChP from the 4th ventricle was sampled by laser-capture microdissection.

### RNA-Seq

Total RNA from individual samples of ChP from mice of Ctrl, SCNx and KO groups was purified by RNeasy Micro Kit (Qiagen, Germany) following the manufacturer’s instructions, treated with On-Column DNAse I digestion set (Sigma) for 15 min at 37 °C, and then quantified by spectrophotometry (NanoDrop, ThermoFisher, USA) and fluorimetry (Qubit, ThermoFisher, concentration range 14–49 ng/µl). RNA integrity was tested by capillary electrophoresis (Agilent, USA) with average RIN = 9.1 (7.3 for frozen KO samples). Samples of purified total RNA (*n* = 24 for Ctrl, SCNx; *n* = 2 for KO separately) were processed at the Functional Genomics and Bioinformatics Service Laboratory of the Institute of Molecular Genetics (Prague, Czechia) as follows: rRNA depletion, polyA RNA enrichment, and library preparation using KAPA mRNA HyperPrep Kit (Roche, Switzerland) or Smarter Stranded totRNA-seq low input kit (Takara, Japan; for KO samples); sequencing using Illumina (USA) NextSeq 2000 P3 (50 cycles, SE). Bioinformatics was performed using the nf-core/rnaseq (v3.10.1, 10.5281/zenodo.1400710) workflow pipeline. Reads were mapped to the latest Genome Reference Consortium Mouse Build 39 (Mm_39_104_nf310); all samples passed quality control (MultiQC v1.13); read counts and transcripts per million (TPM) were calculated by Salmon and Stringtie. Only genes with average TPM ≥ 1 / sample (i.e., the sum of 12 TPM values in 12 samples in each group TPM_sum_ ≥ 12; referred to as filtered background) were analyzed further. Differential expression between all Ctrl and SCNx samples was analyzed by DESeq2 using RNAlysis 3.8 GUI [39].

### Rhythmicity detection

To identify circadian cycling genes with medium-to-high amplitude, three different algorithms were used – eJTK [40–42], ARSER [43] and BIO_CYCLE [44, 45]. After examining the dependence of number of cycling genes on Benjamini-Hochberg (BH) false discovery rate (FDR)-adjusted score (FDR corrected empirical P value in case of eJTK, FDR adjusted Q value in case of ARSER and BIO_CYCLE) of all three algorithms, the rhythmicity threshold was set at below 0.4. To detect phase of the cycling genes, BIO_CYCLE variable LAG was used; to predict relative amplitude and avoid its masking by highly expressed medium amplitude genes, minimum TPM values of each gene were set to 1 before using BIO_CYCLE to calculate AMPLITUDE variable from the normalized TPM values. Rhythmic genes in Ctrl group were divided to six clusters according to their profiles by K-means clustering in RNAlysis 3.8.

### Enrichment, statistics and visualization

Genes were divided into subsets using either DESeq2, rhythmicity threshold or K-means clustering and submitted to over-representation analysis (ORA, implemented in WebGestalt toolkit [46]) against filtered background, showing top ten enriched terms with BH FDR < 0.1 (0.25 in case of differential expression between pooled Ctrl and SCNx samples). Cytoscape v.3.10 was used to detect and visualize protein-protein interactions (STRING database) significantly enriched (FDR < 0.05) against filtered background. Bar plots were prepared in Prism (Graphpad, USA), other plots in seaborn + matplotlib, statistical comparisons (Wilcoxon signed rank test, Kruskal-Wallis and Dunn’s test) were performed in Prism, scipy or statsmodels Python packages.

### In vivo injections

*mPer2*^*Luc*^ mice with successfully ablated SCN (checked as described above; *n* = 6) were arrhythmic in constant darkness for 14 days. At 12:00 (corresponded to ZT5 on the previous LD cycle) on day 1, they were subjected to intraperitoneal injection of dexamethasone (SCNx + Dex; 0.15 mg/ml Dex in PBS solution, 0.2 ml per mice, approx. 1 mg/kg, Merck) in darkness using night vision goggles with minimal handling. The injections were repeated at the same time on day 2 and day 3. On day 3, the animals were sacrificed 1 h after injection and organotypic ChP explants were prepared as detailed below. Since the injection is a stressful procedure that increases endogenous glucocorticoid levels, the effect of Dex on the ChP clock was compared with the clock parameters in naïve SCNx mice.

### Organotypic explant preparation and bioluminescence recordings

*mPer2*^*Luc*^ mice from Ctrl (*n* = 3), SCNx (*n* = 3) and SCNx + Dex (*n* = 6) groups were sacrificed by rapid cervical dislocation between 12:00 and 13:00, corresponding to CT5-6 of the Ctrl group with intact SCN. ChP explants were dissected from the 4th ventricle as described previously [21]. The explants were immediately placed onto Millicell Culture Inserts (Merck) inside a 6-well plate containing 1 ml of air-buffered recording medium (DMEM supplemented with 100 U/ml penicillin, 100 µg/ml streptomycin, 1% GlutaMAX, 2% B27 supplement (ThermoFisher) and 0.1 mM D-Luciferin (Biosynth, Switzerland)). The bioluminescence traces were recorded in a motorized Luminoview LV200 luminescence microscope (Olympus, Japan) fitted with an autofocusing LUCPLFLN20X objective (Olympus), a stage top incubator set to 37 °C/100% humidity (Tokai HIT, Japan) and a water cooled ImageEM X2 EMCCD camera (Hamamatsu, Japan), with an exposure time of 9.5 min and electromagnetic gain 250. Six explants in a plate were imaged every 1 h for 120 h by CellSens Dimensions (Olympus) and exported as 16-bit TIFFs. Cosmic rays were removed by pixelwise subtraction of consecutive images. Whole ChP explants were manually traced in Fiji ImageJ and algorithmically subdivided into 4684–6434 approximately cell-sized regions of interest (ROIs). Data were exported as mean intensity signal and XY coordinates and analyzed after detrending and denoising by a custom modified Python script based on per2py package [47].

## Results

### SCN lesion results in downregulation of endoplasmic reticulum and cation transport genes in choroid plexus in vivo

ChP samples collected from Ctrl and SCNx C57BL/6J mice were sequenced on the Illumina NextSeq 2000. We obtained 41.6–48 million reads uniquely mapped to the mouse genome per each wild type ChP sample. After exploring TPM cross-sample correlation and distribution (Fig. S2A, B), we further analyzed all genes with average TPM ≥ 1 / sample that were assigned a unique gene ID (*n* = 13,077).

Successful isolation of ChP samples without contamination from surrounding cerebral tissue was confirmed by the result that among the chromosomal genes with the highest expression were those coding for well-known ChP-enriched secreted proteins (supplemental Table S1), such as *Ttr* (transthyretin, which transports thyroxine from the bloodstream to the brain), *Enpp2* (autotaxin, responsible for the production of lysophosphatidic acid in extracellular fluids), *Clu* (clusterin, extracellular chaperone), *Igf2* (insulin-like growth factor 2) and *Igfbp2* (insulin-like growth factor-binding protein 2) [30, 31], as well as ChP markers such as *Folr1* (folate receptor alpha [48]).

We used DESeq2 to explore differential expression between the Ctrl and SCNx groups. Only 11 genes were significantly differentially expressed between the groups (FDR < 0.05, max. log fold change ± 0.3) when all time points were analyzed together; 174 genes with FDR < 0.25 (Fig. 1E) were submitted to over-representation analysis (ORA), which revealed several significantly enriched Gene Ontology (GO) terms related to Endoplasmic reticulum (ER), ER membrane, Golgi complex and cation transport (Fig. 1F). Among the downregulated genes was *Slc22a8* (organic anion transporter 3, *Oat3*), a previously described organic transporter responsible for organic anion distribution in CSF [49]. As the overall differences between the whole Ctrl and SCNx groups were minor, we performed time-resolved transcriptomic analysis.

### Circadian transcriptional rhythms in ChP depend on intact SCN in vivo

To identify circadian cycling transcripts, we took advantage of two-day sequential sampling in constant darkness. We employed three previously described algorithms, each based on different rhythmicity detection method – eJTK, ARSER and BIO_CYCLE (see methods for details). We empirically set alpha value for FDR-adjusted Q-values of all three methods to 40% based on exploring how they influence the number of identified cycling genes in both groups (Fig. 1G). This allowed us to detect considerable number of rhythmic transcripts in the Ctrl group (*n* = 732, Fig. 1H, I left) while simultaneously minimizing the number of low amplitude and/or false positives in the SCNx group (*n* = 27, Fig. 1H, I right). This resulted in a large number of false positives with clearly non-circadian variation in transcript levels. Our data underscore the need for careful use of rhythmicity prediction algorithms, ideally together with arrhythmic or randomized control.

When ranked by relative amplitude, the four top scoring genes in Ctrl group were clock genes *Nr1d1/Rev-Erbα*, *Ciart/Chrono*, *Arntl/Bmal1* and *Dbp*. Additionally, all other known clock genes were identified as rhythmic, with the exception of *Cry1* and *Rora* (which were below the rhythmicity threshold, see Fig. 4A and discussion), and *Rorb* and *Bmal2* (which were not expressed in ChP). Most rhythmic genes peaked around the beginning of the subjective night at CT10-14 or at CT18-20 (Fig. 1J, left), while very few rhythmic genes peaked in the first half of subjective day between CT0 and CT9.

After surgical destruction of the SCN, ChP’s global transcriptome displayed almost no detectable high or medium amplitude circadian oscillations (Fig. 1K). Although we cannot rule out the possibility that widespread shallow oscillations persisted, a comparison between the same genes in Ctrl and SCNx groups showed a dramatic and highly significant (Wilcoxon signed-rank test, *P* = 8 × 10^− 114^) decrease in amplitude after SCN lesion (Fig. 1L, left). Of the genes identified as rhythmic in the SCNx group only 27 had amplitude marginally higher than that of their Ctrl counterparts (*P* = 0.0039, Fig. 1L, right).

### Circadian transcriptional rhythmicity in ChP dampens in absence of the local clock to the same level as in absence of the SCN

To assess involvement of the local ChP clock in the circadian rhythmicity, we analyzed the transcriptome of additional ChP samples from mice with genetic knockout of essential clock gene, *Bmal1*, in epithelial cells with motile cilia, including those in ChP (referred to as KO, see Materials and Methods). These samples were collected at two time points: during the day (ZT7) and at night (ZT18). A comparison of the absolute values of normalized day-night differences among the top cycling genes across the three groups suggested (Fig. 1M) that the effects of the targeted genetic disruption of the local clock in ChP were at least comparable to the effects of the SCN lesion on ChP rhythms.

### Circadian regulation, ER protein processing, and histone complex are the main enriched terms among the rhythmic ChP genes

**Fig. 2:**
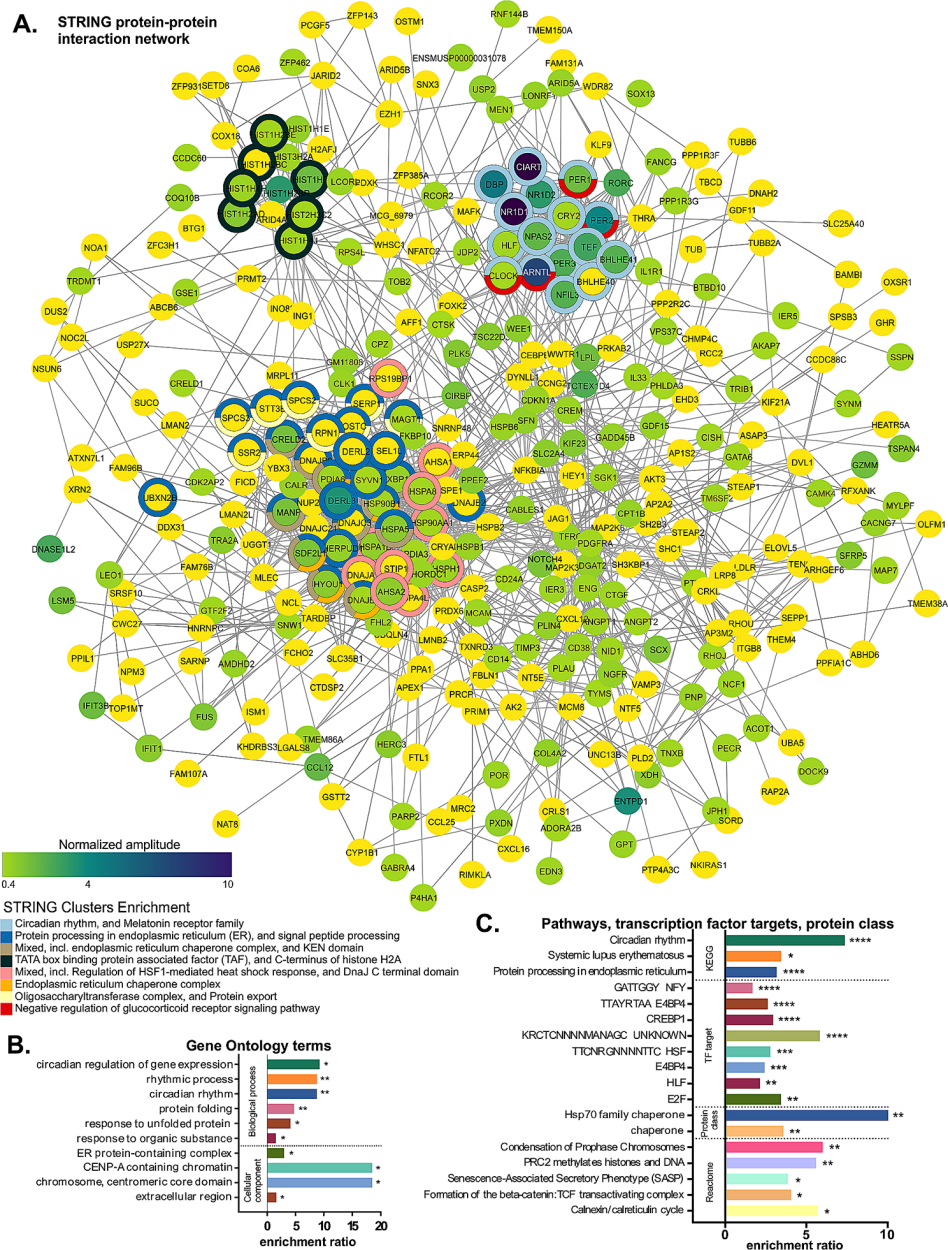
Circadian oscillating genes are dominantly enriched for Circadian rhythm, Endoplasmic reticulum and Histone-related terms. **(A)** Rhythmic genes from Control samples were analyzed for protein-protein interactions using the STRING database. Out of 641 genes recognized by Cytoscape, 494 formed the largest subnetwork, which was further simplified down to nearest neighbors resulting in 345 visualized gene nodes. Normalized amplitude of each rhythmic gene is depicted by color gradient (vivid yellow to blackcurrant, from lowest to highest amplitude). Rhythmic genes functionally annotated via STRING database that were significantly (FDR < 0.05) enriched against the filtered background are marked by color-coded donut charts, see for example genes that are part of the significantly enriched STRING Cluster “Circadian rhythm” in light blue (see legend). Note the three main subnetworks formed by clock genes, chaperones and histones. **(B)** Rhythmic genes from Control samples were submitted to ORA against filtered background. Bar plot of significantly enriched GO Biological process and Cellular component terms showing fold enrichment with asterisks denoting FDR. **(C)** Bar plot of significantly enriched pathways (KEGG, Reactome databases), Transcription factor (TF) targets and PANTHER Protein Classes. * FDR < 0.05, ** FDR < 0.01, *** FDR < 0.001, **** FDR < 0.0001

We employed ORA, analyzing all rhythmic genes against filtered background, to characterize the circadian transcriptome of ChP in Ctrl mice. First, we visualized the gene network using the STRING protein-protein interactions database in Cytoscape (Fig. 2A). The majority (77%) of the identified rhythmic genes formed a single large network, with three major subnetworks enriched in Circadian rhythm (clock genes), ER Protein processing (mainly ER chaperones, see details below), and C-terminus of histone H2A (mainly histones and associated proteins). Moreover, related GO (Fig. 2B) and KEGG (Fig. 2C) terms were significantly enriched. Chaperone Protein class, ER and histone-related Reactome terms were similarly enriched (Fig. 2C). Finally, we identified several enriched transcription factor (TF) target sequences (Fig. 2C) – among them, known circadian regulators, such as E4BP4/NFIL3, CREBP1/ATF2 and HLF, as well as the heat shock factor (HSF).

### Time-specific clustering of rhythmic ChP processes

**Fig. 3:**
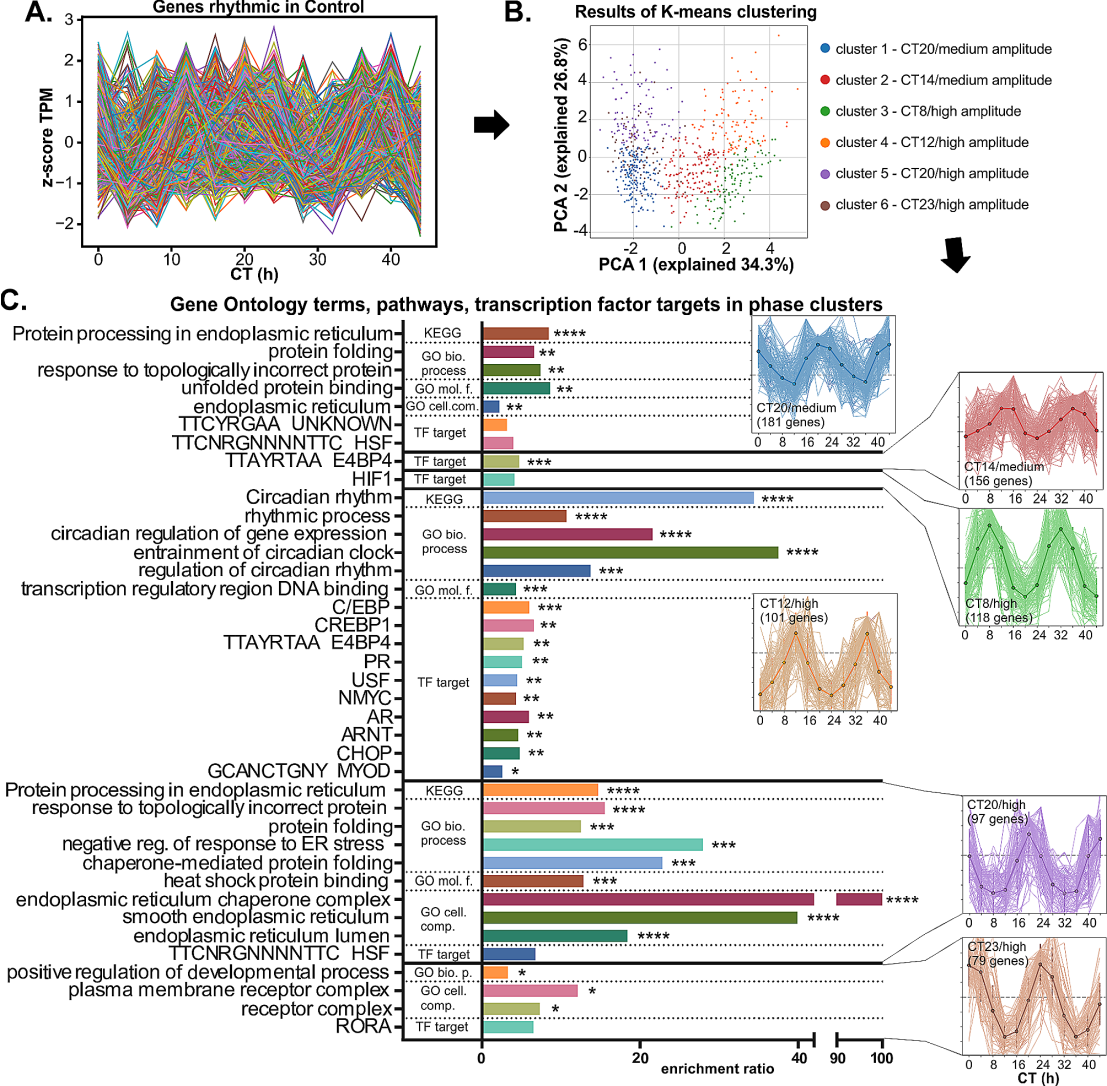
Rhythmically controlled ChP functions cluster in specific phase windows, mainly during subjective night. **(A)** Normalized expression of all rhythmic genes from Control samples. **(B)** Rhythmic genes from Control were divided to 6 groups using K-means clustering. Plot shows principal component analysis with color codes used to differentiate individual clusters 1–6, which correspond to phase-aligned genes. **(C)** Genes from each cluster were subjected to ORA against filtered background. Bar plot of significantly enriched GO terms, TF targets and KEGG pathways showing fold enrichment with asterisks denoting FDR. On the right are traces of normalized expression of each rhythmic gene in each analyzed cluster

To identify the time of day when specific rhythmic ChP functions are activated in Ctrl mice, we used K-means clustering to categorize all rhythmic genes (Fig. 3A) into six distinct phase/amplitude clusters (Fig. 3B and C, right), each containing between 79 and 181 genes.

The largest cluster peaked around CT20 with medium amplitude (CT20/medium) (Fig. 3C, blue). This, together with similarly phased cluster peaking at CT20 but with a high amplitude (CT20/high) (Fig. 3C, violet), was enriched for HSF transcription targets, the Protein processing in ER KEGG term, the ER GO cellular component, and the Protein folding GO biological process terms. The cluster CT20/medium was enriched for the Unfolded protein binding GO molecular function term, while the cluster CT20/high for the Heat shock protein binding GO molecular function term. A STRING protein-protein interactions analysis showed that the cluster CT20/medium was enriched for Protein processing in ER, Oligosaccharyltransferase complex and Chaperone complex. In contrast, the cluster CT20/high was enriched for the Unfolded protein response, Chaperone complex, Nucleosome and other STRING cluster terms (Fig. S2C).

The cluster that peaked around CT14 with medium amplitude (Fig. 3C, red) was enriched for E4BP4 (NFIL3) transcription targets, among them immune response genes involved in NF-κB signaling (Fig. S2C).

The cluster that peaked at CT8 with high amplitude (Fig. 3C, green) was enriched for HIF1 (hypoxia inducible factor) transcription targets and the LRRC8/pannexins STRING cluster term (volume-regulated anion channel subunits, Fig. S2C).

The cluster that peaked at CT12 with high amplitude (Fig. 3C, orange) contained clock and clock-controlled genes regulated via E-box. It was enriched for the Circadian rhythm KEGG and STRING terms, Rhythmic process and related GO biological process terms, the Transcription regulatory region DNA binding GO molecular function term and several TF target terms, including C/EBP, CREBP1, E4BP4 and ARNT. It also contained genes involved in WNT, glucocorticoid, TGF and PDGF signaling (Fig. S2C).

The cluster that peaked around CT23 with high amplitude (Fig. 3C, brown) was enriched for RORA TF targets, with the Positive regulation of developmental process GO Biological process and the Plasma membrane receptor complex GO cellular component terms.

### Rhythmic genes with the highest expression in ChP control endoplasmic reticulum stress response

**Fig. 4:**
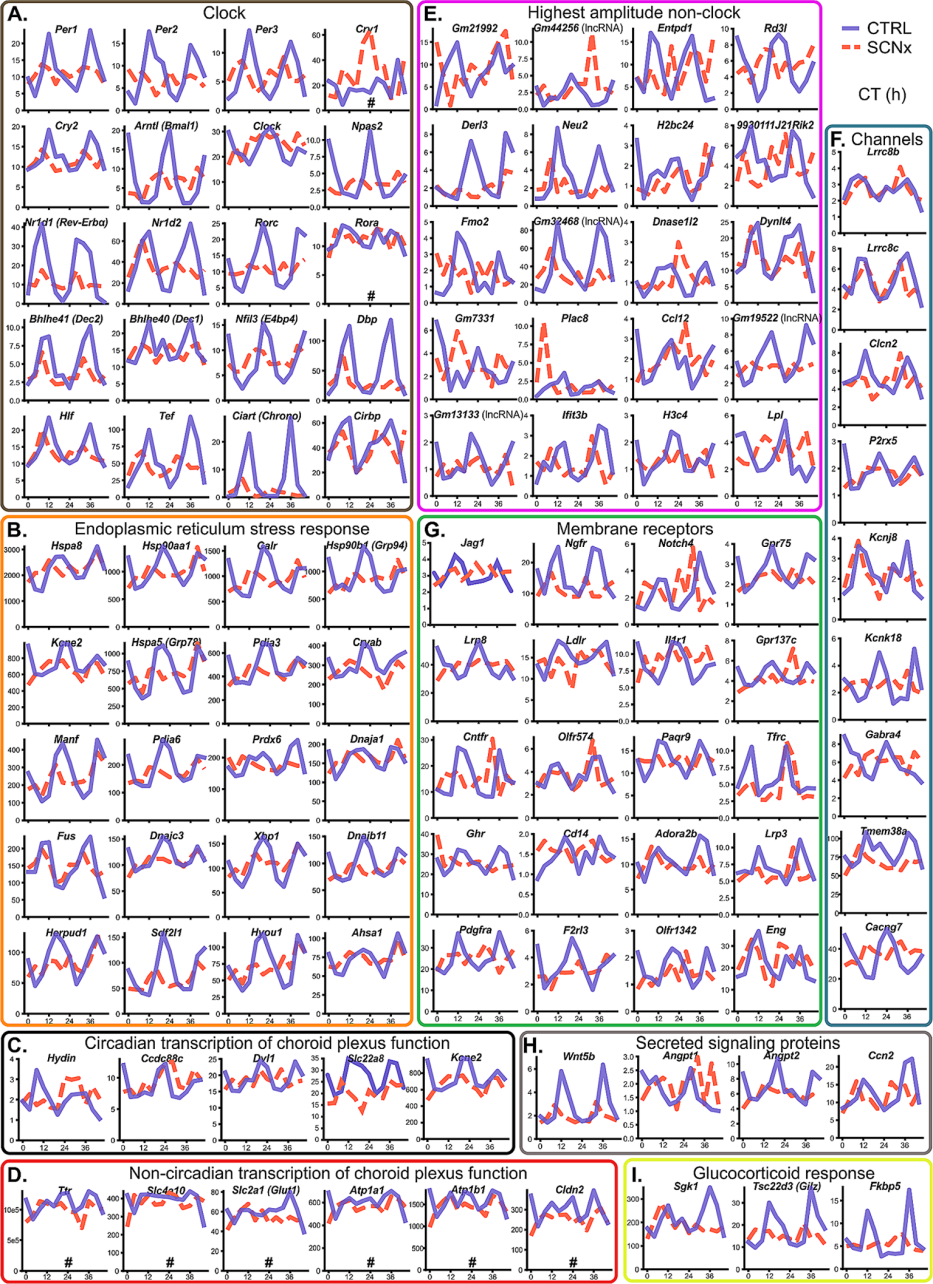
Examples of 48-h traces of rhythmically expressed ChP genes. **(A)** Canonical clock and clock-controlled genes. **(B)** Selected endoplasmic reticulum stress (ERS) response genes. **(C)** Previously described genes with important ChP-specific function that are significantly rhythmic. **(D)** Selected genes with important ChP-specific function that are not significantly rhythmic on mRNA level. **(E)** Non-clock genes with highest circadian amplitude. **(F)** Rhythmic genes coding ion channels. **(G)** Rhythmic genes coding membrane receptors. **(H)** Rhythmic genes coding signaling proteins secreted to CSF. **(I)** Evidence for rhythmic response to glucocorticoid receptor activation. Circadian time (CT) in hours is on X-axis, transcripts per million (TPM) on Y-axis. Control samples blue full line, SCNx samples red dashed line. Genes that are not significantly rhythmic in Control are marked with #

In ChP of Ctrl mice, the six most highly expressed rhythmic genes (*Hspa8, Hsp90aa1, Calr, Hsp90b1/GRP94, Kcne2*, and *Hspa5/GRP78/BiP*) and more than half of the remaining top 50 most highly expressed rhythmic genes (including transcription factor *Xbp1* and its target *Sdf2l1* [50]) play a role in endoplasmic reticulum stress (ERS) response or unfolded protein response (representative genes from this group are shown in Fig. 4B). We visualized the corresponding KEGG pathway (Protein processing in Endoplasmic reticulum, Fig. S3), showing that Chaperone-mediated protein recognition, Folding and ER-associated degradation (ERAD) processes, as well as the main regulatory complexes XBP and CHOP contain genes under circadian regulation. All of them belong to clusters peaking at CT20 with medium or high amplitude (Fig. 3C, S2C), with the exception of *Fus* (fused in sarcoma) which peaked at CT12. The gene codes an RNA-binding component of hnRNP complex involved in splicing that actually induces ERS only in its mutated form commonly expressed in several types of cancer and amyotrophic lateral sclerosis [51, 52]. Interestingly, several other splicing related genes (e.g., *Lsm5*, *Cwc27*; Fig. S5) followed similar expression pattern as *Fus*, suggesting that both RNA and protein processing is rhythmic but with distinct timing.

### Genes regulating WNT signaling and CSF composition are rhythmically regulated

We identified circadian cycling transcripts of genes previously described to play specific roles in ChP functions in Ctrl mice (Fig. 4C). Here, we list only a few examples.

*Hydin* (hydrocephalus-inducing protein homolog) is a gene required for ciliary motility. Its mutation leads to the accumulation of CSF within the ventricles [53]). *Ccdc88c* (coiled-coil domain containing 88 C, or *Daple*) is a gene whose mutation causes congenital hydrocephalus [54]. Its product interacts with and negatively regulates the cytoplasmic WNT signaling transducer disheveled, whose transcript *Dvl1* was also significantly rhythmic in ChP. Moreover, the gene for the Frizzled ligand (*Wnt5b*, see Fig. 4H) was rhythmic with high amplitude, as were other genes involved in both canonical (*Porcn, Sfrp5, Bambi, Nkd2*) and non-canonical WNT pathways (*Nkd2, Nfatc2*, see Fig. S4).

Another group of rhythmically expressed genes is involved in the modulation of CSF composition. The product of *Slc22a8*, OAT3, controls the organic anion distribution in CSF [49], including prostaglandins (reviewed in [55]). *Kcne2* (Potassium Voltage-Gated Channel Subfamily E Regulatory Subunit 2) regulates the CSF ion composition [56]. All of these genes lost circadian expression in the SCNx mice; in the case of *Slc22a8*, this was accompanied by significant downregulation (DESeq2, FDR < 0.05; Fig. 1E). Additionally, other genes potentially involved in CSF composition, such as genes coding for ion channels and secreted signaling proteins, are listed in the following subchapter.

On the other hand, some genes critical for CSF production, such as *Ttr* (Fig. 4D), *Folr1*, *Enpp2*, *Clu* (Table S1), were either constitutively expressed or their mRNA variation was below the rhythmicity threshold. We also found non-circadian expression of several genes previously hypothesized [19] to be responsible for diurnal changes in CSF volume and composition (Fig. 4D). These include the ion cotransporter *Slc4a10*, *Glut1*/*Slc2a1*, ATPase subunit genes, tight junction genes such as *Cldn2*, and aquaporins *Aqp1/4/11*, suggesting that their potential circadian regulation might need to occur at the level of protein abundance or activity, as was recently demonstrated for *Ttr* [32].

### Many ion channels, membrane receptors, and secreted signaling proteins are rhythmically regulated

With the exception of known clock genes, we identified many previously uncharacterized, highly rhythmic genes in ChP of Ctrl mice (Fig. 4E). Among the highest amplitude non-clock transcripts (Fig. 4E) were several lncRNAs and pseudogenes, as well as genes coding for components of the ERAD pathway (*Derl3*), various enzymes (*Entpd1*, *Neu2*, *Dnase1l2*, *Fmo2*, *Lpl*), and histones (*H2bc24*, *H3c4*).

We identified at least 9 genes for ion channels or their regulatory subunits (including K^+^, Ca^2+^, and Cl^−^ channels that may affect the CSF composition, Fig. 4F) and more than 20 genes for various membrane receptors and their interacting partners. These include genes for NOTCH4 receptor and its ligand JAGGED1 (*Jag1*), ciliary neurotrophic factor receptor (*Cntfr*), interleukin 1 receptor (*Il1r1*), platelet-derived growth factor receptor A (*Pdgfra*), nerve growth factor receptor (*Ngfr*), growth hormone receptor (*Ghr*), and tumor growth factor receptor β subunit (*Eng*; Fig. 4G), all exhibiting medium or high amplitude rhythms.

We detected high-amplitude expression of genes coding for secreted signaling proteins and known mitogens (Fig. 4H), such as above-mentioned *Wnt5b*, two angiopoietin genes, and cellular communication network factor 2 (*Ccn2*). We also found evidence of rhythmic activation of the glucocorticoid receptor (GR) signaling pathway, with its markers such as *Gilz* (*Tsc22d3*) tracking the peak in blood glucocorticoids during first half of the night (Fig. 4I).

Many genes involved in immune response (including interleukin 1 and lipopolysaccharide receptors, Fig. 4G; NF-κB signaling components *Nfkbia* and *Nkiras1*, suppressor of cytokine signaling *Cish* and others, Fig. S5), cell cycle regulation (including G2 checkpoint kinase *Wee1* and p21^CIP1/WAF1^ G1 checkpoint inhibitor gene *Cdkn1a*, Fig. S5), splicing (Fig. S5), and vesicle trafficking (Fig. S5) were rhythmically expressed with high amplitude. We also found rhythmic transcripts for at least 15 histones and histone-associated proteins (Fig. S5), 4 cadherin genes coding transmembrane adhesion proteins, including recently reported circadian cadherin *Cdh3* (Fig. S5; [32]), at least 20 transporters potentially involved in circadian regulation of CSF composition (including *Abcb6* and *Magt1*), 8 G-proteins and G protein-coupled receptors (Fig. S6).

Unlike ERS genes, which all clustered around CT20 (Fig. S2C, 4B), the phase of these functional genes was much more varied. For example, WNT, NOTCH and GR signaling genes clustered around CT12, indicative of E-box driven expression, while interleukin 1 receptor gene was expressed in antiphase and E4BP4/NFIL3 targets governing immune response clustered around CT4. Similarly, genes for K^+^ and Ca^2+^ channels were expressed in different phases.

Together, our data demonstrate broad circadian rhythmicity of ChP transcriptome involved in diverse time-specific functions, with a very high emphasis on nighttime expression, which is, nevertheless, highly dependent on systemic rhythms driven by the central circadian pacemaker in the SCN.

### SCN lesion disturbs circadian clock in individual ChP cells

**Fig. 5:**
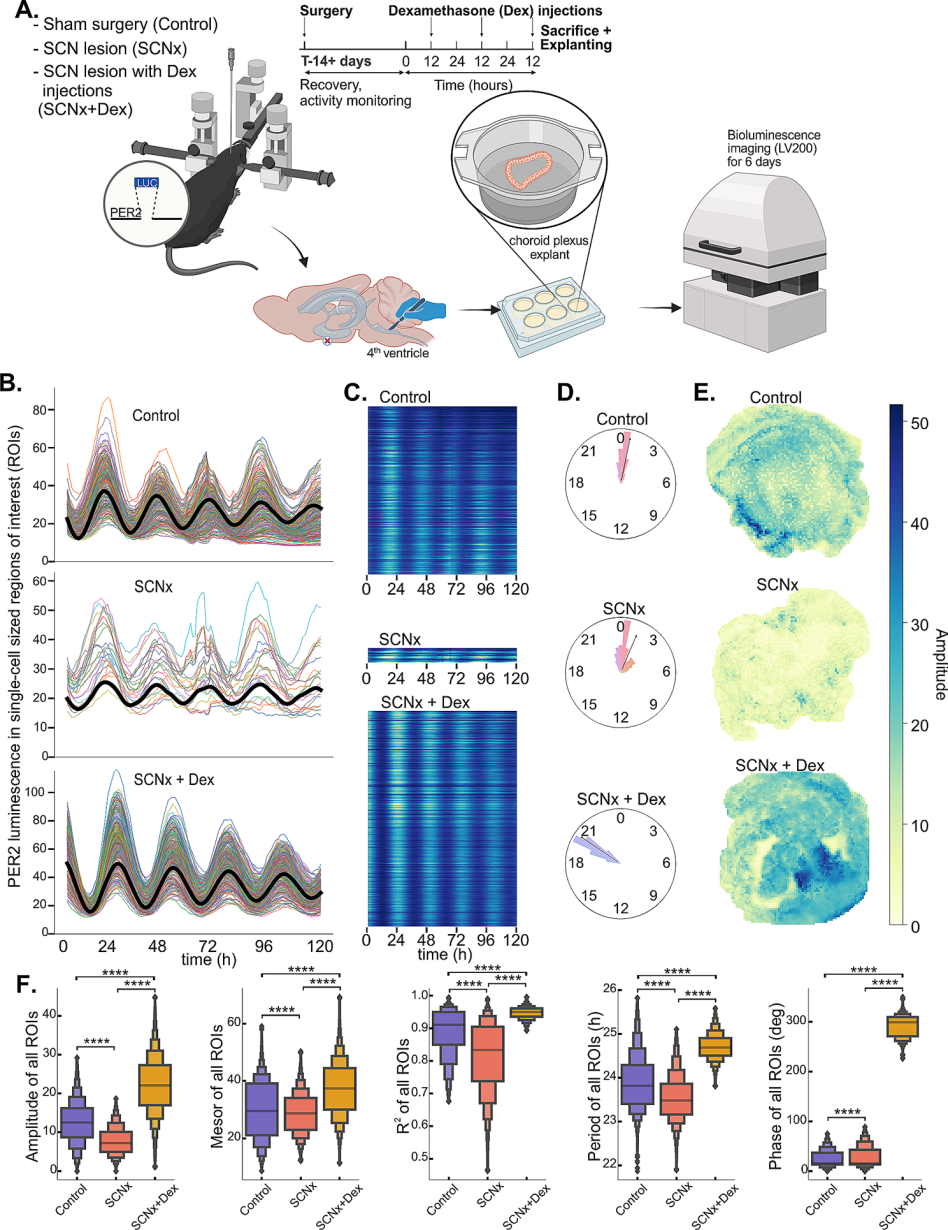
ChP explants from SCNx *mPer2*^*Luc*^ mice have compromised circadian rhythms ex vivo, which can be restored by a repeated in vivo injection of glucocorticoid analog dexamethasone. **(A)** Cartoon depicting the workflow and the three experimental groups – mice with sham surgery (Control), SCN lesion (SCNx) and SCN lesion with three consecutive dexamethasone injections (SCNx + Dex); created with BioRender.com. **(B)** Circadian PER2::LUCIFERASE rhythms in approximately single-cell sized regions of interest (ROIs) across a whole representative explanted ChP from either Control, SCNx or SCNx + Dex mouse recorded for 5 days ex vivo using Luminoview LV200 (Olympus). Only traces that could be fitted with cosine curve with period between 18–35 h and goodness of fit R^2^ > 0.97 are shown. Average cosine fit is shown in black. **(C)** Heatmap of the same data, showing that there were many more rhythmic cells with R^2^ > 0.97 in Control than in SCNx explants and Dex efficiently restored the number of rhythmic rois. **(D)** Polar histogram of all rhythmic ROIs in Control, SCNx and SCNx + Dex explants with calculated Rayleigh vector showing the mean phase. **(E)** Positional heatmaps showing spatial distribution of all rhythmic ROIs across representative explants from Control, SCNx and SCNx + Dex mice. **(F)** Violin plots showing comparison between circadian parameters (from left: amplitude, mesor, R^2^, period, phase) of all rhythmic ROIs (excluding outliers outside 3 standard deviations) across individual ChP explants from Control (blue), SCNx (red) and SCNx + Dex (orange) mice (Kruskal-Wallis with Dunn’s post-hoc test, asterisks depict significant difference between each group on *P* < 0.0001, number of mice and individual explants *n* = 3–6 / group, number of analyzed ROIs *n* = 4684–6434 / explant)

The massive dampening of ChP circadian transcriptome oscillations in response to lesion of the SCN (Fig. 1) could be attributed to a loss of rhythms at the single-cell level, a loss of synchrony among cellular oscillators, or, more likely, a combination of both. To address this, we explanted ChP from *mPer2*^*Luc*^ mice subjected to sham surgery (Ctrl; *n* = 3) or SCN lesion (SCNx; *n* = 3; SCNx was verified as detailed above) to monitor PER2-driven bioluminescence in individual cell-sized regions of interest (ROIs) of the whole ChP tissue using a luminescence microscope (LV200, Fig. 5A). The recording started immediately after harvesting the explants in the presence of luciferin and lasted for 5 days. While explants from both Ctrl and SCNx mice were rhythmic, the overall amplitude and PER2 protein luminescence levels were noticeably reduced in the SCNx explants (Fig. 5B-E, upper vs. middle plot). Analysis of individual ROIs confirmed that the overall rhythm in SCNx explants was compromised. Setting the rhythmicity threshold of cosine curve goodness of fit at R^2^ = 0.97 revealed dramatically fewer high-amplitude oscillating ROIs in the SCNx compared to the Ctrl explants (Fig. 5B, C). The phases of the ROI rhythms in SCNx explants were more dispersed (Fig. 5D), and their amplitudes were lower on average and more uniform (Fig. 5E) across the whole explant. A statistical comparison (Fig. 5F, blue vs. red) between all recorded ROIs across Ctrl and SCNx explants showed that the amplitude, mesor (average PER2 level), and goodness of fit were significantly reduced in the SCNx explants (Mann-Whitney, *P* < 0.0001). In addition, the period was shorter (*P* < 0.0001), and the phase was more spread out (*P* < 0.0001) in the SCNx than in the Ctrl explants. Thus, the presence or absence of SCN in vivo has lasting effects on ChP circadian cycling in static culture ex vivo, despite the fact that local clocks still drive the rhythms in ChP cells.

### In vivo dexamethasone injections increase the amplitude of the ex vivo rhythms in SCNx ChPs

In a last step, we attempted to uncover a possible mechanism of how the SCN controls the ChP clock. Based on our previous data [21], we hypothesized that glucocorticoids and subsequent GR activation play a role as the major SCN-driven signal that controls the ChP clock. Indeed, our transcriptomic analysis showed that GR activation was highly rhythmic in the Ctrl ChP, but the rhythmicity was lost due to SCNx (Fig. 4I). SCN ablated arrhythmic *mPer2*^*Luc*^ mice (*n* = 6) kept in constant darkness received injection of corticosterone analog dexamethasone at 12.00 h (corresponding to ZT5 of the previous LD cycle) on three consecutive days (SCNx + Dex), and we analyzed the rhythms in ChP explants harvested 1 h after the last injection (Fig. 5A). We hypothesized that if the GR signaling pathway is involved in ChP clock entrainment in vivo, Dex injections should be able to at least partially rescue the number of rhythmic ROIs ex vivo. Indeed, analysis of ex vivo rhythms in single cell-sized ROIs, as described above, showed that both the number of rhythmic ROIs (Fig. 5B, C, lower plot), their amplitude, mesor (average level of PER2 luminescence over the course of the analysis), goodness of fit (R^2^) and period (Fig. 5E, lower plot and Fig. 5F, orange) increased dramatically in SCNx ChP after Dex injections (Kruskal-Wallis with Dunn’s test, *P* < 0.0001), even above and beyond the rhythm in Ctrl group. Moreover, the ChP clock was phase advanced by 5–9 h when compared with Control or SCNx group (Fig. 5D, F), consistent with the advanced timing of Dex injections relative to the endogenous corticosterone surge. Overall, the data provide evidence that the SCN-regulated glucocorticoid signaling is one of the possible drivers of circadian rhythms in ChP.

## Discussion

The lack of time-resolved map of ChP transcriptome has hindered understanding of the circadian regulation of ChP functions. Our results provide deep insight into the broad circadian regulation of cellular processes in the mouse ChP that underlie the ChP specific functions. We show that the ChP rhythms are highly dependent on the presence of functional SCN clock. Despite the fact that the ChP clock exhibits self-autonomous rhythmicity in conditions of long term static ex vivo culture [18, 21, 22, 57], we show that in vivo, the clock and majority of the rhythmic processes dampen in the absence of rhythmic signals from the SCN. While we do not rule out the persistence of low amplitude rhythms in the absence of the SCN clock [58], the extent of the rhythm dampening seemed comparable to that we found after genetic deletion of the local ChP clock. Although we could not perform detection of rhythmicity in the KO animals due to their low numbers, its absence is to be expected since in spite of presence of partial *Arntl* transcript, the resulting BMAL1 protein is not functional due to excised exon 8 [18]. The effect of SCNx on dampening the local ChP clock persists for days in such a static ex vivo culture and is not simply a result of desynchronized cellular oscillators but appears at the single-cell level. Finally, we provide evidence that the SCN-controlled GR signaling may provide one of the entraining signals to clocks in the ChP cells. The high and previously unexpected dependence of the circadian rhythm in ChP on the central clock [18] points to its high vulnerability to circadian misalignment resulting from unhealthy lifestyles in humans [59]. Therefore, understanding processes driven by ChP clock is of utmost importance.

Our results point to at a unique feature of the mouse ChP clock, which is based on robust rhythmic expression of all canonical clock genes with the remarkable exception of *Cry1*. *Cry1* mRNA shows circadian variation in the SCN [60, 61] and peripheral tissues such as liver, lung, heart, spleen, kidney, intestine, muscle, adipose tissue and macrophages [62–67] due to multiple E-boxes, D-boxes and ROREs in its promoter [68]. While circadian expression of *Cry1* and *Cry2* is important for the proper function of TTFL [69, 70], it is not strictly necessary [68, 71, 72]. Moreover, rhythmic *Cry2* can likely fully complement the circadian role of *Cry1* in ChP. Although *Cry1* has recently been shown to be arrhythmic in the rat hippocampus [73], to our knowledge, ChP is the only tissue with a robust clock and arrhythmic *Cry1* expression. Nevertheless, the presence of possible diurnal variation cannot be ruled out, as a recent publication comparing the rat ChP transcriptome between one daytime and one nighttime time point found differential expression [74].

By far, the most enriched circadian transcripts in ChP were the ERS response genes. The ERS response (reviewed in [75]) consists of four signaling cascades: (a) induction of ER chaperones such as HSPA5 (also known as GRP78 or BiP), HSP90B1 (also known as GRP94 or GP96), and CALR (Calreticulin), which promote the folding of unfolded proteins and whose expression is highly rhythmic with a peak around CT20 in ChP; (b) inhibition of protein synthesis; (c) induction of an ER-associated degradation (ERAD) pathway [76] with proteins such as DERL2/3, HERPUD1, UBE2 and HSP90AA1, which promote the ubiquitination and processing of unfolded proteins, and whose expression is again highly rhythmic in ChP; (d) induction of apoptosis by CHOP-mediated inhibition of BCL2 when this system cannot process the unfolded proteins [77]. The expression of the CHOP gene *Ddit3* falls just below our rhythmicity threshold (Q_*Ddit3*_ = 0.4061), while the upstream regulatory TF *Xbp1* is significantly rhythmic. Importantly, one of the main triggers of ERS is excessive protein synthesis and related metabolic stress [78]. Fame et al. [32] recently showed that translation and protein secretion in mouse ChP may be diurnally regulated as it exhibits high levels during the dark phase (ZT14) and low levels during the light phase (ZT2). Our transcriptomic data analyzed in samples collected over two cycles in constant darkness show that ERS response is the highest during the second part of the subjective night with the peak of chaperone mRNA at CT20 (corresponding to ZT20 on the LD cycle). Hence, it may prepare ChP for the incoming stress caused by massive accumulation of secreted proteins such as thyroid hormone transporter transthyretin (*Ttr* alone constitutes about 17% of total protein coding non-mitochondrial mRNA present at any moment in ChP cells, Table S1) after several hours of ongoing translation. Precisely scheduled ERS may also help to rapidly shut down the translation when needed and fine-tune the timing of circadian secretion. The ChP clock thus not only regulates the circadian secretome of the CSF [32], but may also protect ChP from associated cytotoxic damage by precise timing of the ERS response [79].

The overt ex vivo rhythms of cultured cells [80], many explanted peripheral [33, 81], and brain tissues [82] dampen over time due to gradual decoupling of individual cellular oscillators. The ChP clock maintains high amplitude rhythms ex vivo over a longer interval [18] but the mechanism of how the intercellular synchrony is maintained in the static culture of extra-SCN tissue is unclear. Recently, a rhythmic ER-Golgi-Endosome secretory pathway was reported to contribute to intercellular synchrony in dense cultures of U-2 OS cells [83]. Interestingly, we found that the TGF-β coreceptor gene *Eng* is also rhythmically expressed in ChP. Our results suggest that paracrine coupling involving TGF-β may be involved in achieving high synchrony among individual ChP cells, and thus help to maintain these rhythms in the absence of external rhythmic signals. Under in vivo condition, multiple mutually synchronized rhythmic cues may complement paracrine signaling and contribute to maintaining the high amplitude ChP rhythms. Intriguingly, we show that surgical ablation of the SCN to mice dampened the high amplitude PER2 bioluminescence oscillations monitored in ChP ex vivo, which indicates that SCNx may compromise synchrony among the entraining signals.

As an important outcome of our study, we identified circadian expression of a large set of protein-coding genes involved in multiple ChP-specific functions, such as CSF production, defense against immune challenge, metabolite clearance from the brain, and disease-related processes, as discussed below.

The CSF secretion shows diurnal variation with a peak during the night hours in humans [23, 84]. Although the involvement of the ChP clock in CSF production has been hypothesized [18], its mechanism has been questioned due to the absence of daily variation in the expression of the water channel, aquaporin 1 (*Aqp1*) [20]. Our results confirm the absence of circadian rhythm in *Aqp1* and other aquaporin genes at the mRNA level. Nevertheless, we identified other markers of the CSF production that are under circadian control. For example, tightness of cell adhesion may fluctuate over 24 h because both its upstream (WNT signaling pathway) and downstream (cadherins *Cdh3*, *Cdh18*, *Cdh19*, *Pcdhac2*, but not the tight junction genes such as claudins and occludin) constituents are expressed diurnally. Importantly, the CSF is not a passive ultrafiltrate of the plasma but is actively secreted, for which ChP uses controlled transport of molecules across the cell membrane (reviewed in [85]). We show that genes coding for many actively regulated ion transporters and channels are expressed rhythmically, e.g., transporters for zinc (*Slc39a10*), magnesium (*Magt1*), chloride (*Clcn2*), calcium (*Cacng7*), potassium (*Kcnj8*, *Kcnk18*), and organic anions (*Slco3a1, Slc22a8*). On the other hand, we did not find significantly circadian expression of genes belonging to the SLC12 and SLC4 families of transporters, nor genes coding for subunits of Na+-K + ATPase pump (*Atp1a1*, *Atp1b1-2*), all of which are abundantly expressed in ChP epithelium [31, 85]. Interestingly, transporters for lactate, pyruvate and ketone (*Slc16a6*), phospholipid (*Plscr4*), glucose (*Slc2a9*, *Slc2a4*, but not GLUT1 gene *Slc2a1*), and folate (*Slc19a1*) show significant rhythmicity, suggesting diurnal nourishment of the brain cells via rhythm in the CSF composition. Compared to plasma, the CSF contains about 2-20-fold less free amino acids, with the exception of glutamine, which is nearly equal. This is maintained by the activity of amino acid transporters [86], and we show that expressions of some of them (*Slc6a15* and *Slc6a20b*) exhibit circadian variation. Additionally, ChP cells express rhythmically genes coding for hydrolases, which catabolize fatty acid amides (*Faah*) and glycolipids, glycoproteins, and oligosaccharides (*Neu2*), as well as lipoprotein lipase *(Lpl)*, which breaks down triglycerides from the bloodstream. The circadian regulation of *Lpl* is consistent with a previous report in liver [87]. Altogether, our results reveal circadian regulation of strategic milestones in production and composition of the CSF.

ChP plays a role in forming an immune barrier to separate the blood from the brain, providing protection from infection (reviewed in [88]). Although circadian control of immune processes is well-documented [89, 90], it has not been investigated whether such control is present in ChP. Previous transcriptomic analyses of mouse ChP to identify genes responding to peripheral infection due to repeated LPS treatments revealed the most significant changes in pathways facilitating entry of cells into the CSF fluid and participating in the innate immune response to infection [27]. We found high-amplitude rhythms in the expression of genes for transmembrane receptors for interleukin 1 (*Il1r1*) and for LPS (*Cd14*), several genes for chemokines (*Il33, Ccl12, Ccl25*), for cytokine signaling regulators (*Cish, Cys1, Sh2b3*), and others. Genes coding for components of NF-κB and MAPK signaling pathways (*Nfkbia, Nkiras, Map2k3*) involved in regulation of immune response were also rhythmically transcribed [91]. The results strongly suggest circadian control of multiple processes involved in the immune defense of ChP.

ChP participates in clearing waste, such as metabolites, drugs, and toxins, from the brain. This requires synchronized CSF production by ChP and drainage of interstitial fluid via the sleep-regulated glymphatic system [24, 92], resulting in a non-selective pathway for the removal of solutes and macromolecules via bulk CSF flow. Additionally, ChP cells themselves are able to actively export/degrade both endogenous active biomolecules and xenobiotics. For example, the *Slc22a8* product OAT3 (previously shown to be rhythmic also by others [93]) has been proposed to act as a cerebral clearance pathway for prostaglandins PGE_2_ and PGD_2_ (reviewed in [55]). Since PGE_2_ plays a role in modulating wakefulness [94] and PGD_2_ promotes sleep [95], circadian dysregulation of ChP expressed *Slc22a8* may be an overlooked factor in sleep physiology.

We identified rhythmically expressed genes, which have been associated with developmental processes, such as hydrocephalus-associated genes (*Ccdc88c, Hydin*) [53, 96], and several rhythmically expressed mitogens, which are likely diurnally released into CSF (*Wnt5b, Angpt1-2, Ccn2*) [97–99]. The peak of expression for *Wnt5b*, *Ccn2*, as well as many other genes for receptors and ligands of various signaling pathways, falls at the beginning of the circadian night. Finally, we also found genes related to neurodegenerative diseases. ChP has been implicated in pathologies of Alzheimer’s disease, Huntington’s disease, and frontotemporal dementia [100]. Especially AD has been repeatedly associated with impaired secretory, barrier, transport, and immune function of ChP [88, 101]. In our study, we found circadian regulation of several AD-associated genes. For example, the gene for interleukin 33 (*Il33*), which plays a role in reversing the buildup and preventing the new formation of amyloid plaques in animal AD model APP/PS1 mice [102], *Lrp8os3*, coding for the receptor for AD genetic risk marker apolipoprotein E [103], or *Fhl2*, coding adaptor protein physically interacting with another important AD marker, presenilin 2 [104]. On the other hand, many other genes associated with AD were not rhythmically active (e.g., *Trem2*, and *Mt2*).

In search for a mechanism of how the SCN entrains the ChP clock, we considered the possible role of glucocorticoids based on a synthesis of multiple findings: (a) glucocorticoid release is highly rhythmic [105] as an output signal of the SCN clock [106, 107]; (b) extra-SCN clocks are often sensitive to GR-signaling [82, 108, 109]; (c) our previous data showed that the ChP clock dampens in response to abolishment of the glucocorticoids due to adrenalectomy in vivo and responds to the corticosterone agonist Dex when applied to explants in vitro [21]; (d) our current RNAseq data showed particularly strong rhythmic activation of GR-signaling markers lost in SCNx mice. In this study, we demonstrated that in SCNx mice circadian rhythms in ChP are dampened and their rhythmicity can be restored by intraperitoneal injections of Dex for three consecutive days. The injections not only restored the cellular rhythms in the ChP, but also shifted their phases. The effect of Dex was compared with the rhythm in SCNx mice that were not treated to prevent activation of endogenous glucocorticoids by manipulating the animals, which may potentially provide an additional entraining signal. We showed that circadian variation of corticosterone in vivo could provide the regular humoral signal that maintains the coherent high-amplitude cellular rhythms in ChP. Of course, our results do not exclude the possibility of other rhythmic signals that could also play an important role in entraining the ChP clock. Such mechanisms remain to be discovered.

There are some limitations to our study. (a) We cannot exclude the possibility that we missed low-amplitude and/or low-expressed genes of importance for ChP biology due to we set a threshold for the significance of rhythmicity. (b) The sensitivity of our approach might be limited due to the lack of replicates at each time point and the relatively sparse 4-h sampling interval. However, this was largely compensated by sampling in two cycles as recommended in the Guidelines for Genome-Scale Analysis of Biological Rhythms [110]. For this reason, we focused only on high- and medium-amplitude rhythms with circadian (and not ultradian) periods. (c) We provide temporal but not cellular resolution of the transcriptomic data. However, verification of transcript/cell type can be obtained from data of a recent study by Dani and colleagues [31], in which single-cell RNAseq was performed to compare between developmental stages at one time point. (d) We only analyzed RNA levels (with the exception of real-time PER2 luminescence recording of ex vivo ChP). A recent important study [32] reported diurnal rhythms in ChP at the protein and metabolite levels, though at the lowest possible time resolution (two time points). (e) We only analyzed ChP from the 4th ventricle of male mice to be consistent with our previous study in which we found that the clock is highly sensitive to disrupted environmental cycles [111]. It remains to be clarified whether circadian transcriptomic profiles differ between ChP in the lateral and third ventricle and whether they are sex-dependent. Future studies taking advantage of powerful time-resolved scRNAseq complemented by proteomics and metabolomics may reveal even more details and help us understand the importance of circadian regulation of ChP function.

In summary, we report the first detailed time-resolved transcriptomic study of a brain tissue with an extremely robust circadian clock, ChP of the 4th ventricle, providing the first evidence for many rhythmically regulated processes in this tissue. Furthermore, we show that the ChP clock and its function are highly dependent on the pacemaker clock in the SCN that possibly employs corticosterone rhythm as a humoral entraining signal. Importantly, our study reveals the high sensitivity of the ChP clock to the absence of signals emanating from the SCN in vivo, which in humans may be caused by an unhealthy lifestyle. The extensive role of the ChP in brain homeostasis, together with previously published [18, 32] and current data, provides the basis for the hypothesis that ChP is the main site that transmits circadian disruption to other parts of the brain. Testing this hypothesis could lead to findings with far-reaching implications for considering the function of ChP in neurodevelopment, psychiatric and neurodegenerative disorders, or sleep regulation.

### Electronic supplementary material

Below is the link to the electronic supplementary material.

Supplementary Material 1

Supplementary Material 2

Supplementary Material 3

Supplementary Material 4

Supplementary Material 5

Supplementary Material 6

Supplementary Material 7

## Data Availability

Raw files and technical details about the RNA-Seq data have been deposited in NCBI’s Gene Expression Omnibus and are accessible through GEO Series accession no. GSE243858.
