# Supplementary figures and images for: The circadian clock in the choroid plexus drives rhythms in multiple cellular processes under the control of the suprachiasmatic nucleus
Source: Fluids Barriers CNS. 2024 May 27;21:46. doi: 10.1186/s12987-024-00547-3 (PMC11131265; doi:10.1186/s12987-024-00547-3)

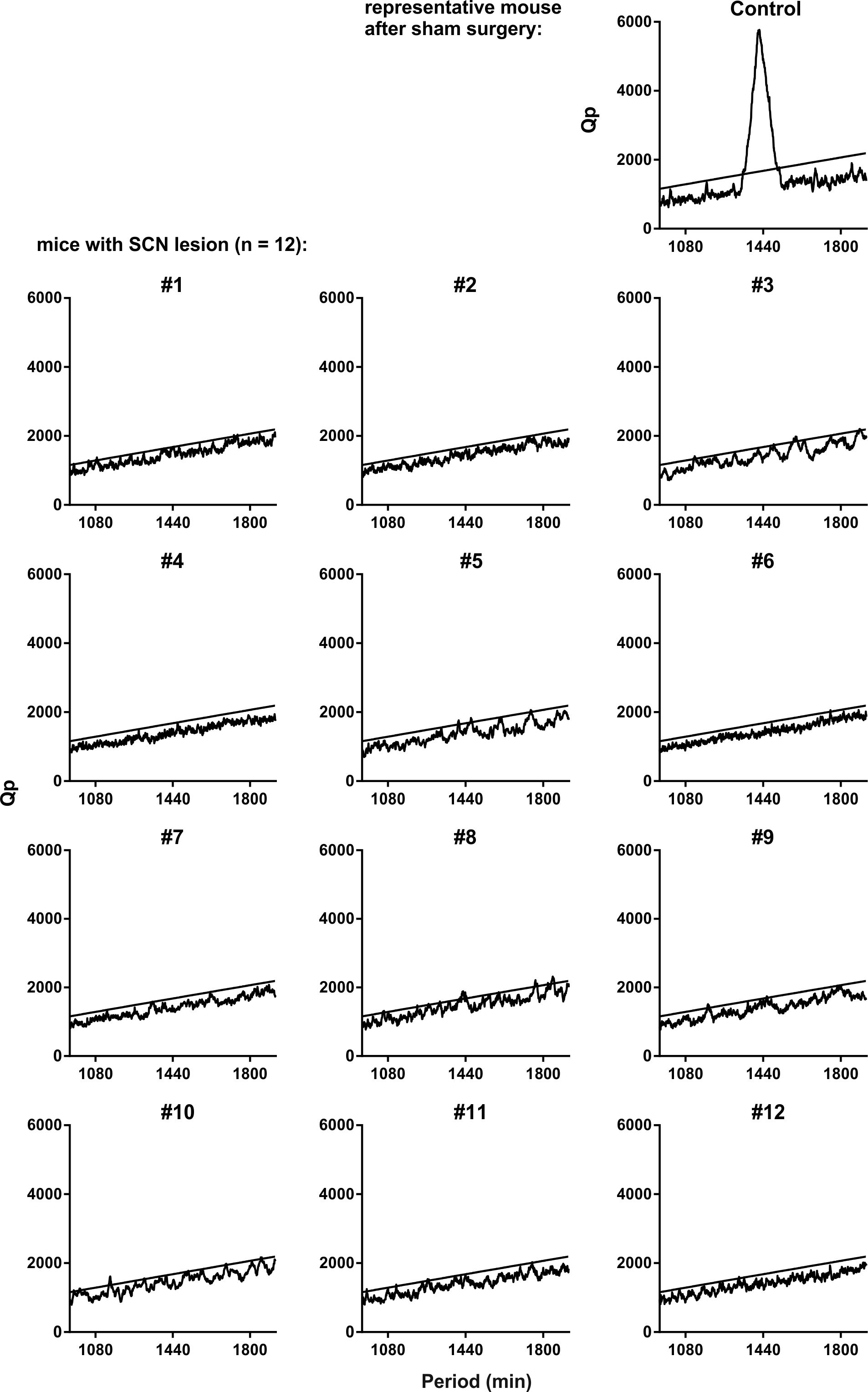

Supplement: Supplementary file 2 — Supplementary Material 2 [file 12987_2024_547_MOESM2_ESM.tif]

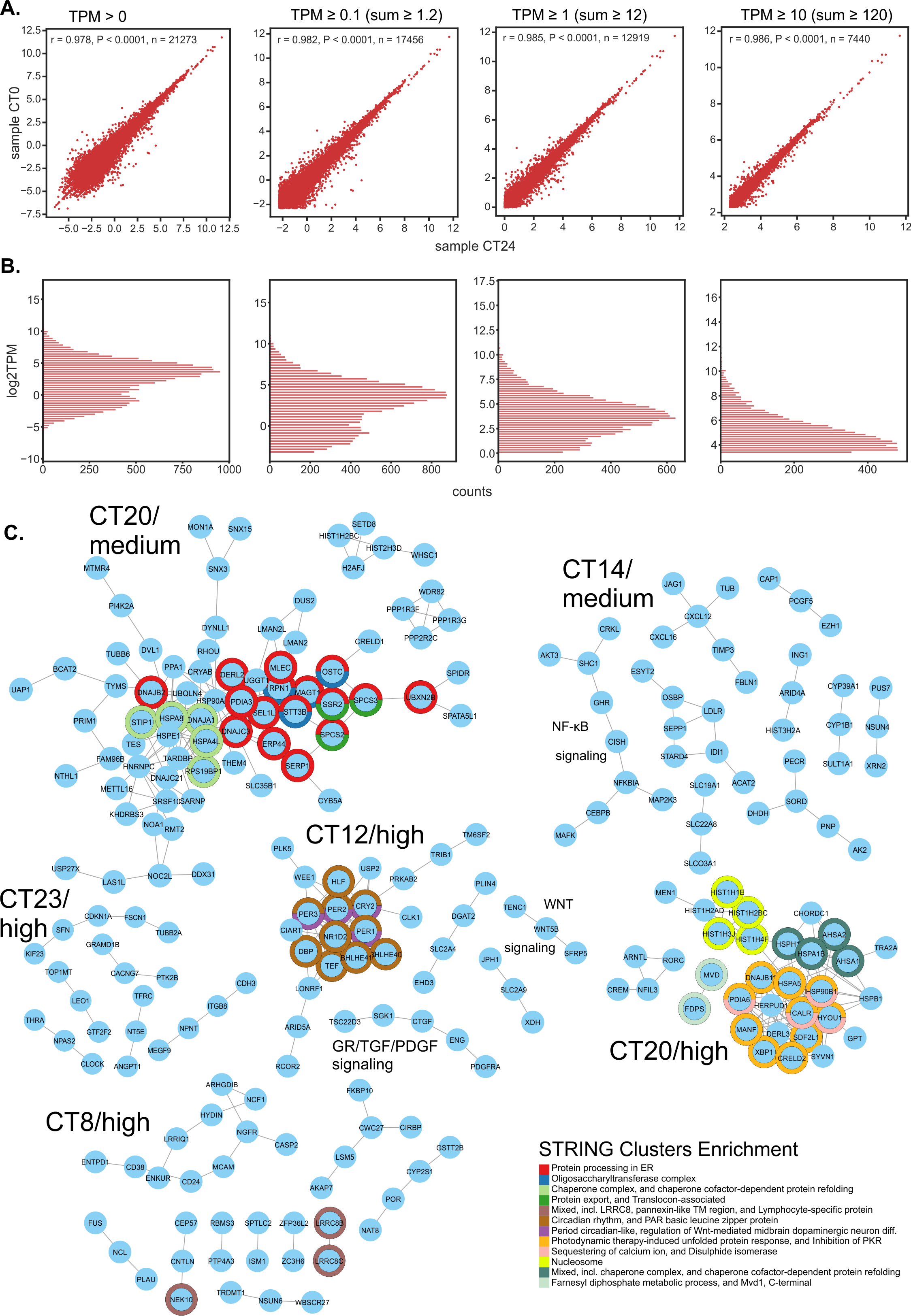

Supplement: Supplementary file 3 — Supplementary Material 3 [file 12987_2024_547_MOESM3_ESM.tif]

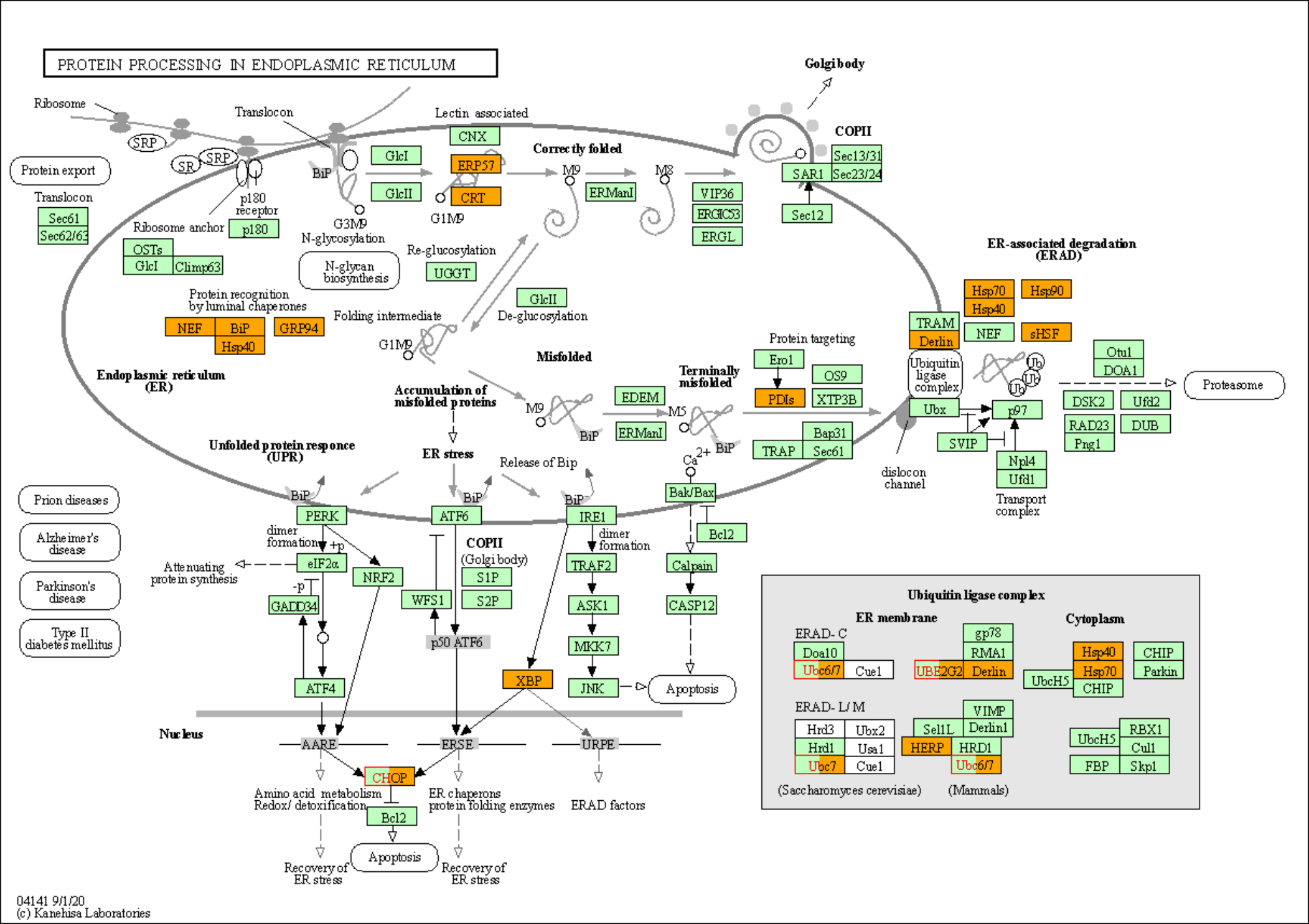

Supplement: Supplementary file 4 — Supplementary Material 4 [file 12987_2024_547_MOESM4_ESM.tif]

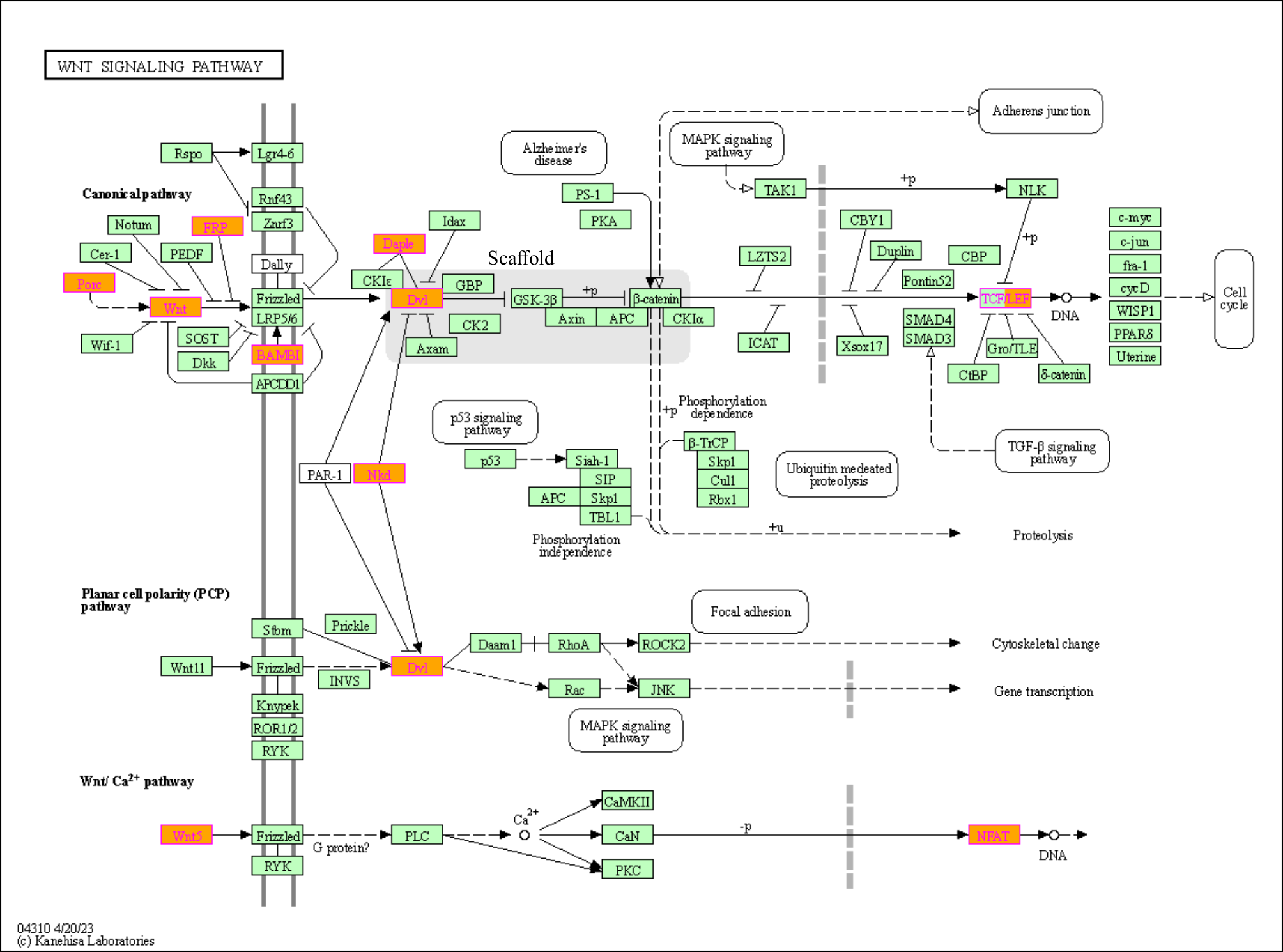

Supplement: Supplementary file 5 — Supplementary Material 5 [file 12987_2024_547_MOESM5_ESM.tif]

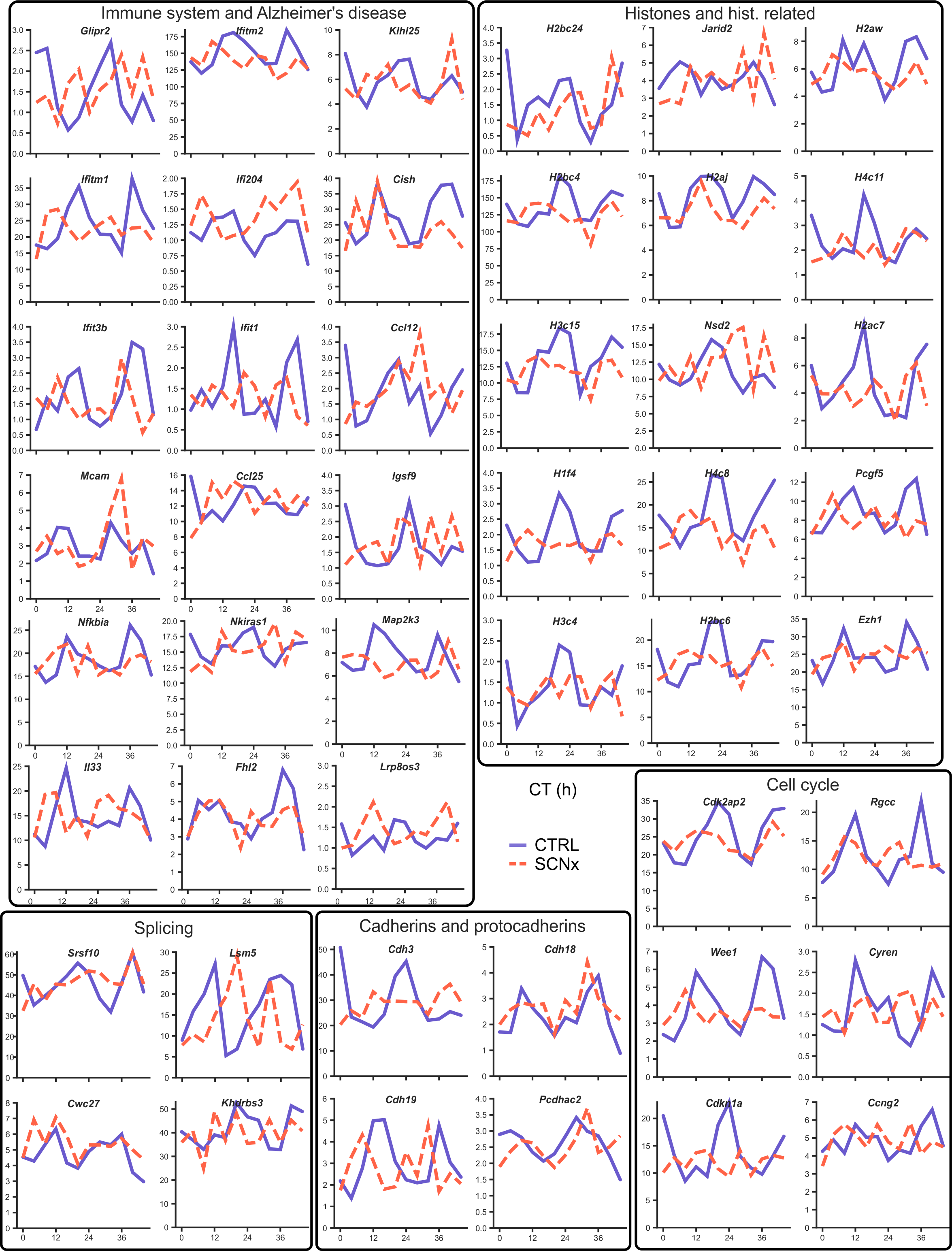

Supplement: Supplementary file 6 — Supplementary Material 6 [file 12987_2024_547_MOESM6_ESM.tif]

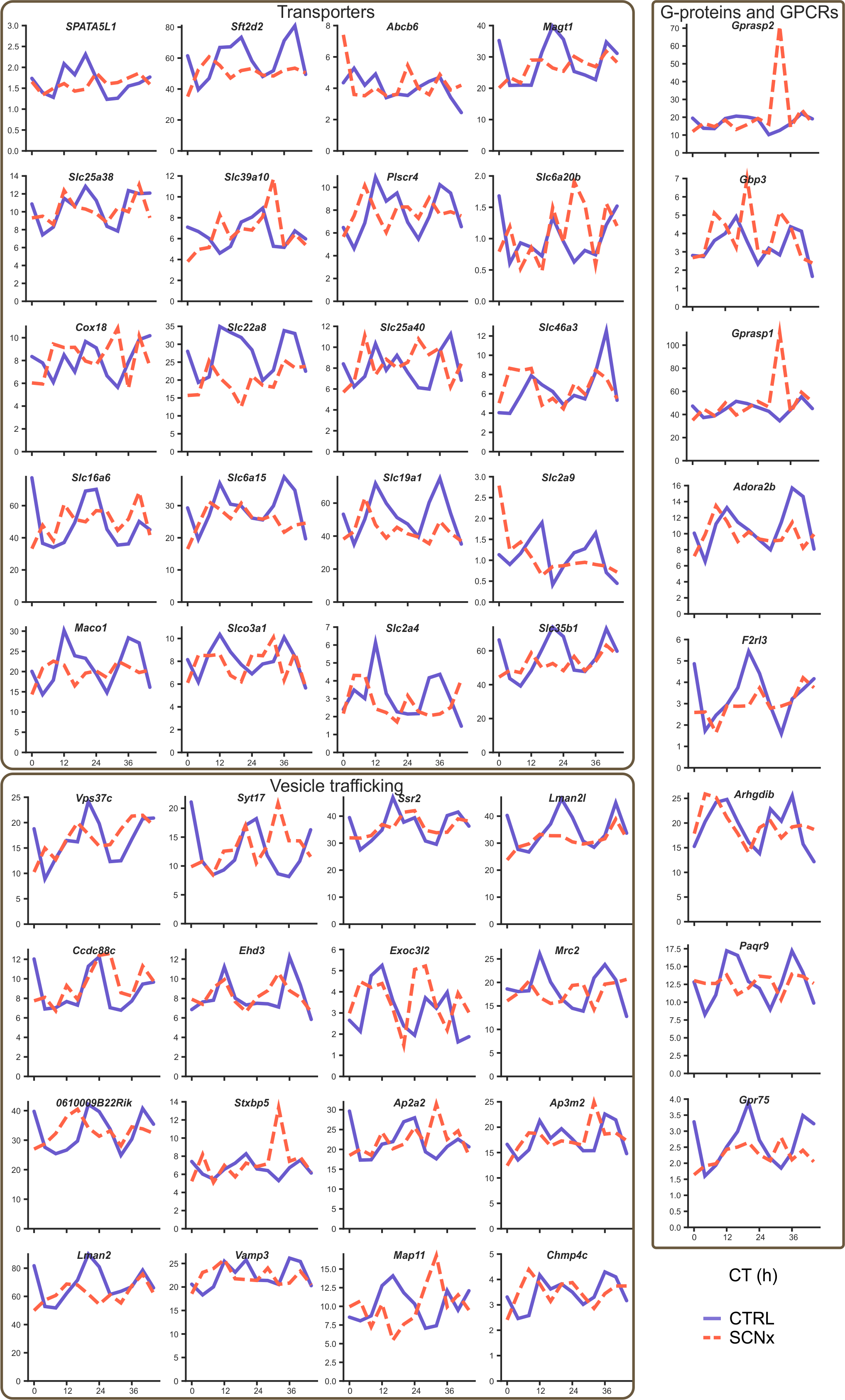

Supplement: Supplementary file 7 — Supplementary Material 7 [file 12987_2024_547_MOESM7_ESM.tif]
